# WRITTEN INFO KEEPS YOU ON TRACK! - Importance of written communication/information in therapeutic adherence

**DOI:** 10.1192/j.eurpsy.2025.1852

**Published:** 2025-08-26

**Authors:** S. Mouta, M. Fonseca, A. Pires, M. Pires, D. Figueiredo

**Affiliations:** 1 Psychiatry and Mental Health Department, Unidade Local de Saúde da Guarda, Guarda, Portugal

## Abstract

**Introduction:**

Therapeutic adherence is defined by the World Health Organization as the degree to which the patient’s behavior corresponds to the recommendations agreed with the healthcare professional. Adequate adherence to pharmacological treatment is essential to achieve therapeutic objectives, but non-adherence rates are high, ranging between 10% and 92%.

**Objectives:**

To highlight the role of written communication/information provided to the patient in the adherence to treatment.

**Methods:**

Non-systematic literature review.

**Results:**

Non-adherence limits therapeutic benefits, compromises the effectiveness of medications and increases the demand for healthcare, representing a major obstacle to the provision of care. One of the factors that contribute to non-adherence is the failure in communication between healthcare professionals and patients, especially with regard to providing clear information about medications.

Since patient educational interventions seem important and effective in improving medication adherence, it is pertinent to adopt more effective ways of communicating and adequately informing patients about the main aspects of the prescribed drugs. Such process can be assisted by written information leaflets.

In this context, studies have revealed that patients appreciate written information to help make decisions about whether or not to take a medication, manage medication intake and interpret symptoms. The benefits and side effects of drugs are generally important information for patients, especially if presented in a legible way, with understandable text, without large volume and without small font size used.

Written instructions can be a useful complement to information transmitted verbally by serving to increase the likelihood that important information can be presented, understood, accepted and remembered by the patient - studies show that patients who received written information better understood their medication, precautions, use instructions, associated side effects, and were more satisfied with the information received, which could contribute to greater therapeutic adherence.

**Conclusions:**

There seems to be a consensus in the bibliography that written information should not replace verbal information. The latter remains a priority, but must be closely associated to written information so that, in combination, its beneficial effects can be enhanced.

**Disclosure of Interest:**

None Declared

